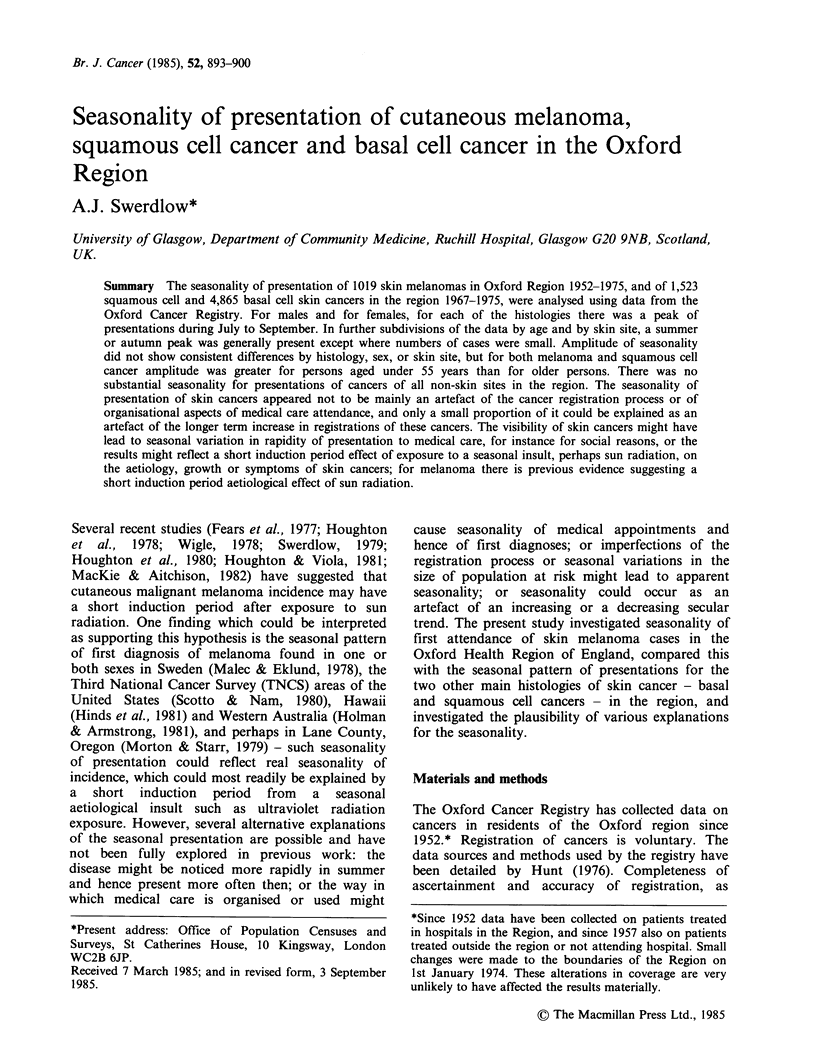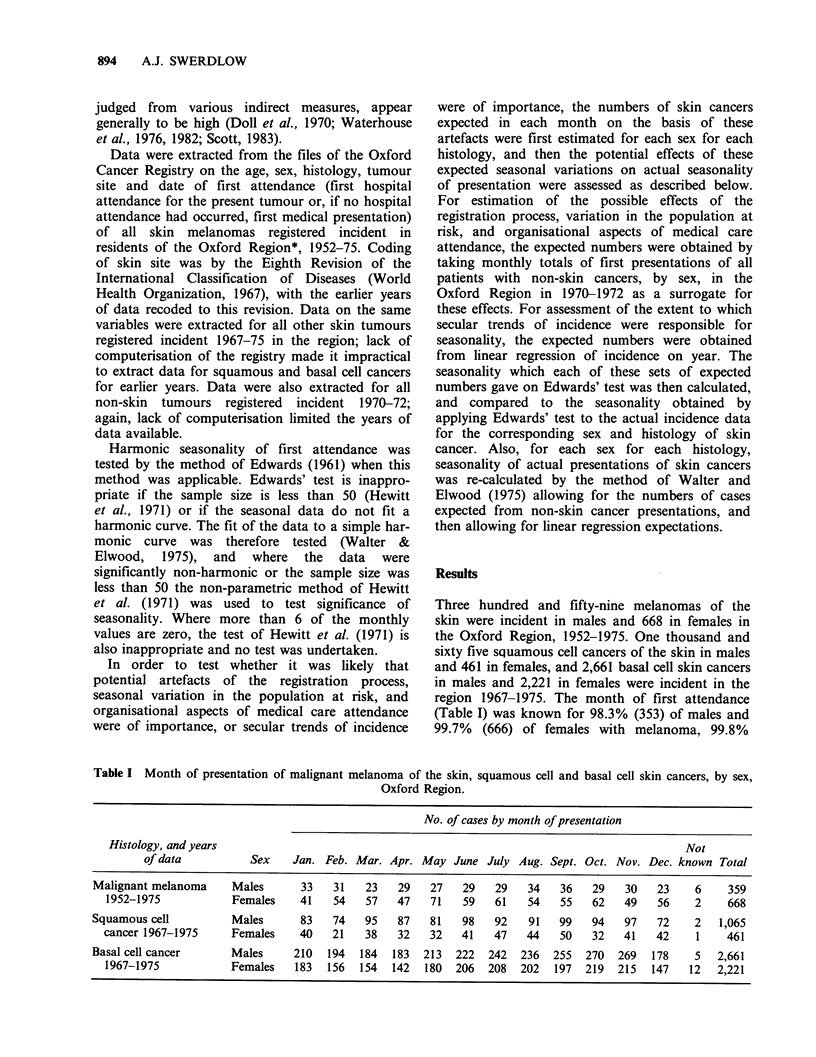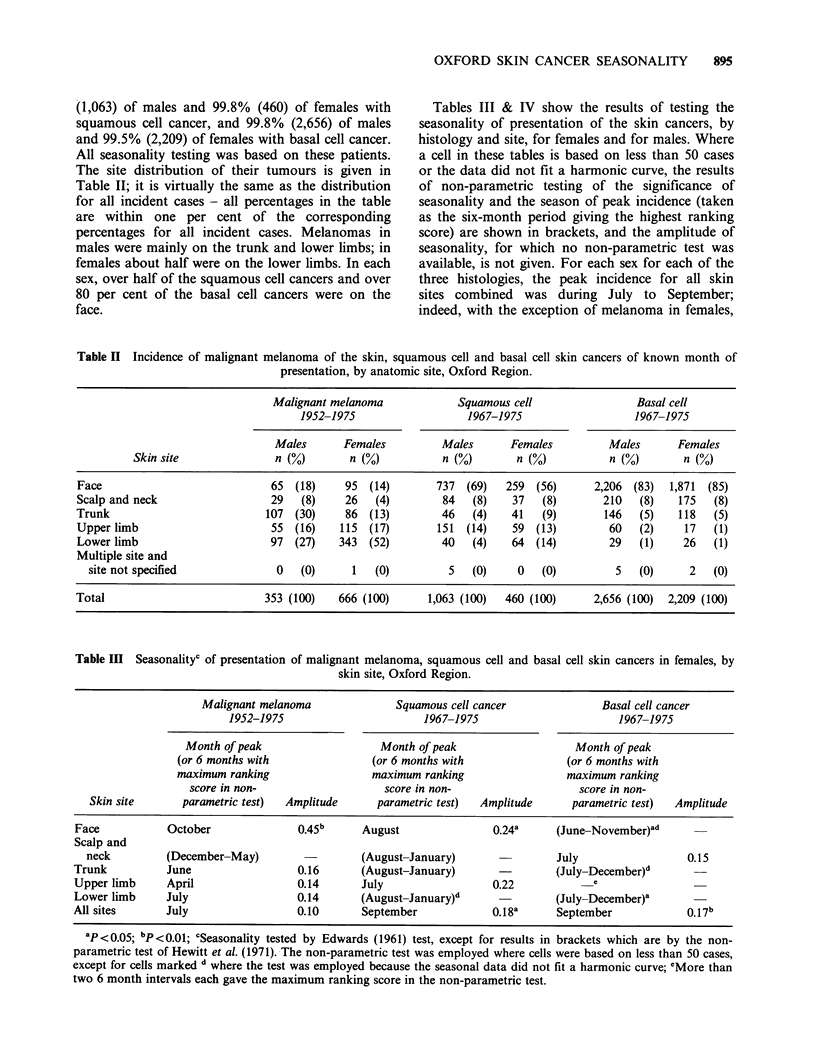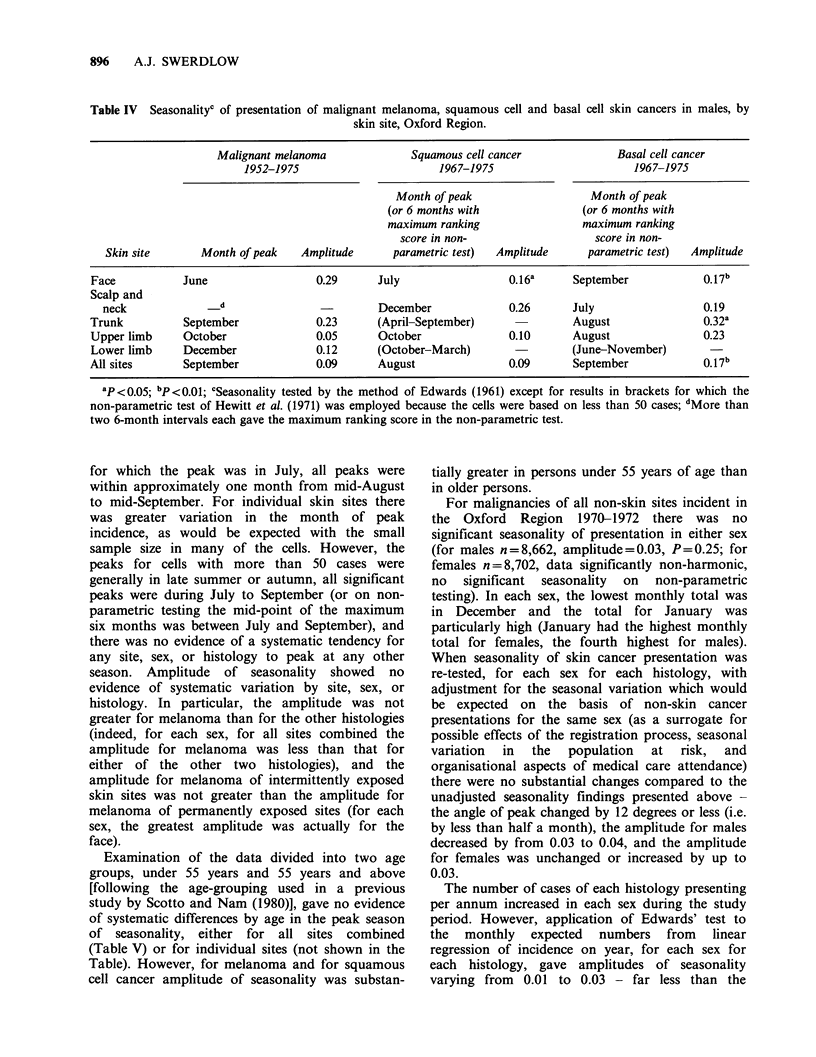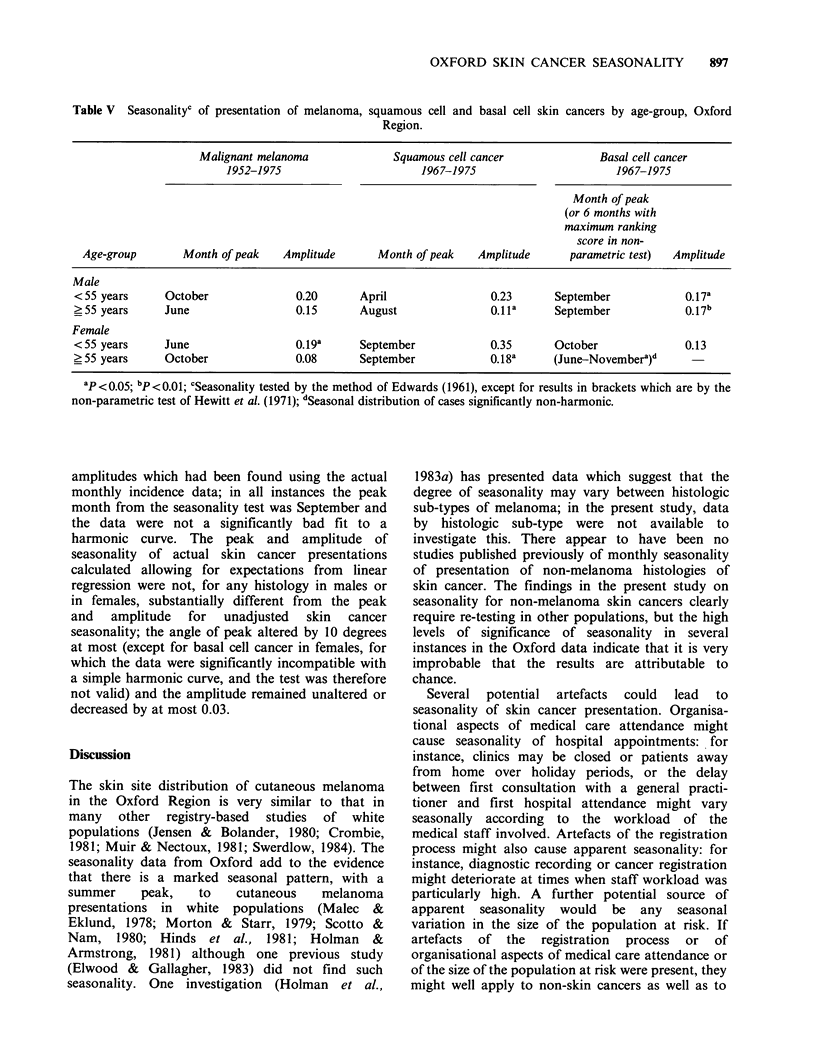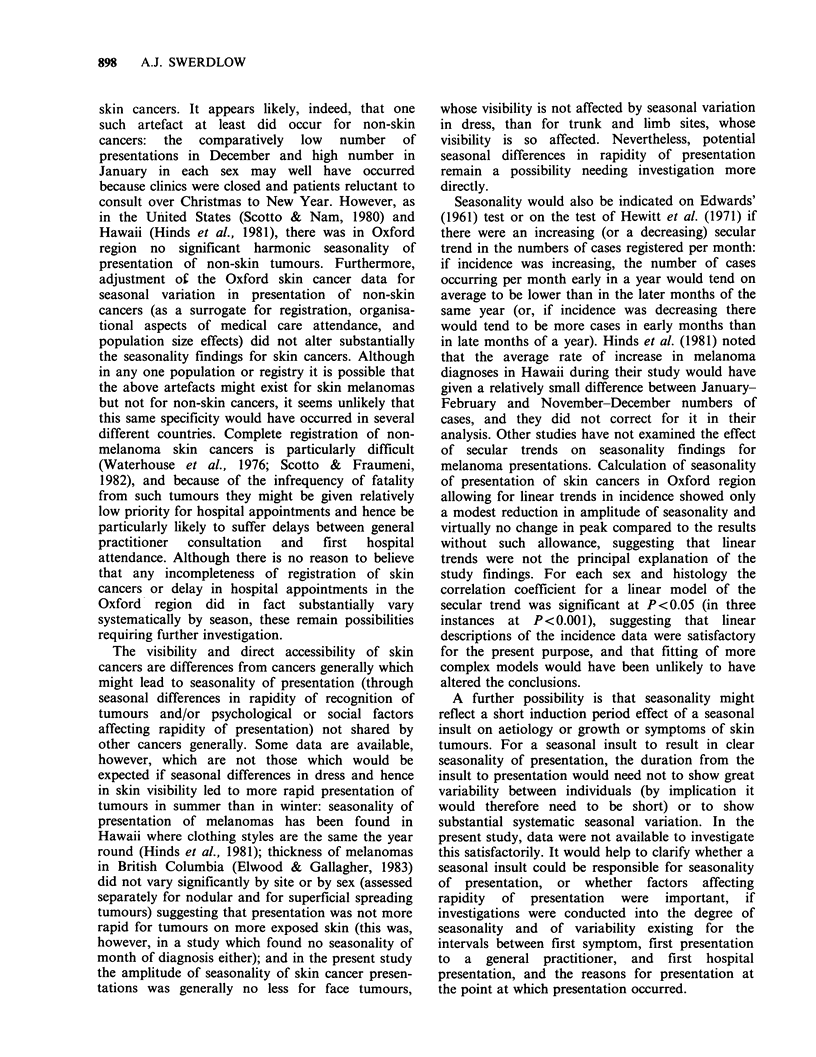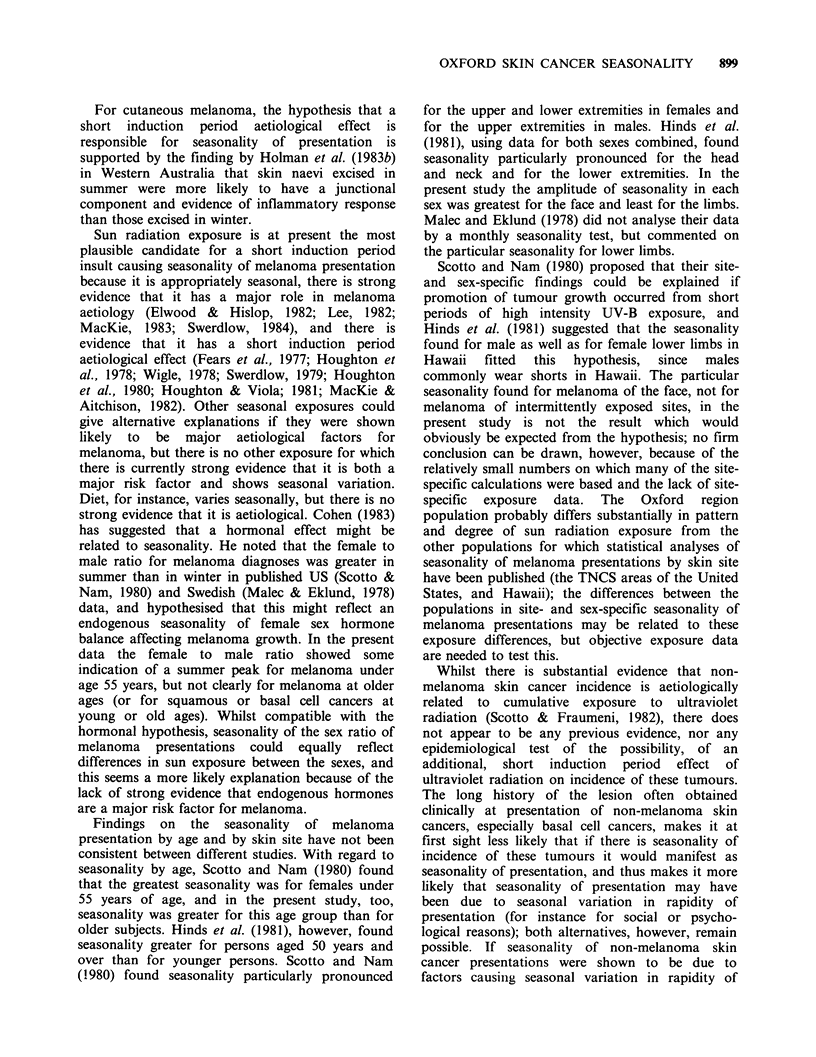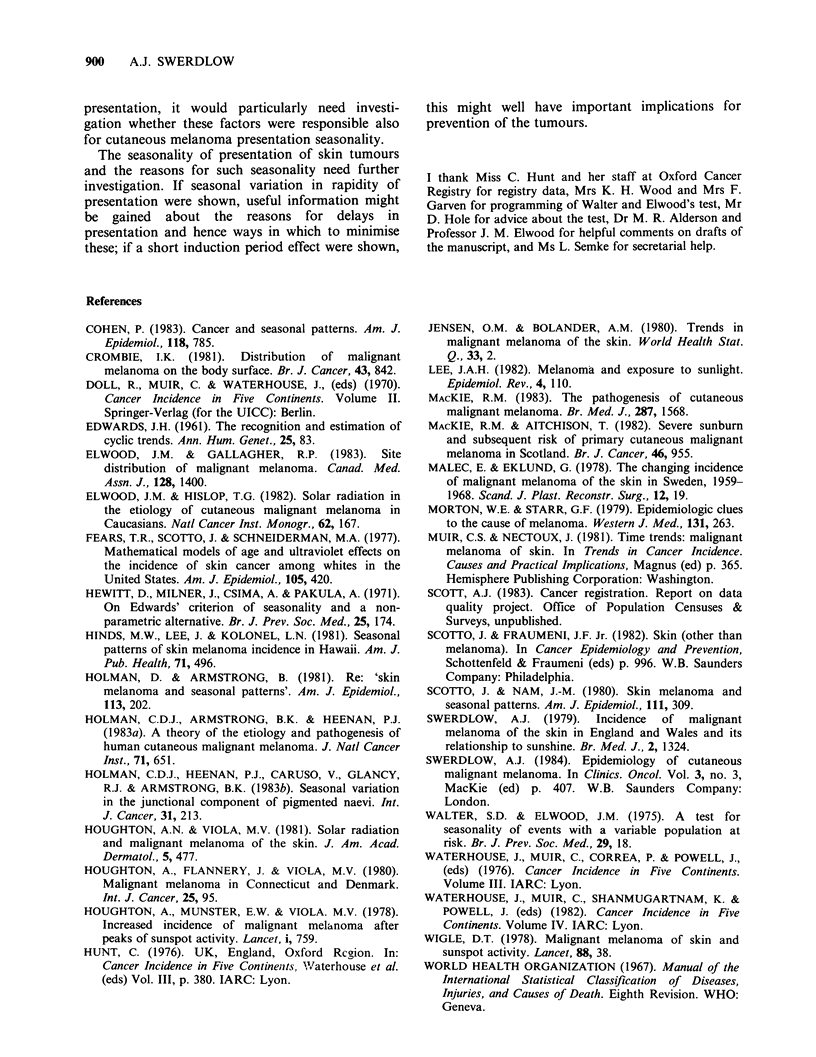# Seasonality of presentation of cutaneous melanoma, squamous cell cancer and basal cell cancer in the Oxford Region.

**DOI:** 10.1038/bjc.1985.274

**Published:** 1985-12

**Authors:** A. J. Swerdlow

## Abstract

The seasonality of presentation of 1019 skin melanomas in Oxford Region 1952-1975, and of 1,523 squamous cell and 4,865 basal cell skin cancers in the region 1967-1975, were analysed using data from the Oxford Cancer Registry. For males and for females, for each of the histologies there was a peak of presentations during July to September. In further subdivisions of the data by age and by skin site, a summer or autumn peak was generally present except where numbers of cases were small. Amplitude of seasonality did not show consistent differences by histology, sex, or skin site, but for both melanoma and squamous cell cancer amplitude was greater for persons aged under 55 years than for older persons. There was no substantial seasonality for presentations of cancers of all non-skin sites in the region. The seasonality of presentation of skin cancers appeared not to be mainly an artefact of the cancer registration process or of organisational aspects of medical care attendance, and only a small proportion of it could be explained as an artefact of the longer term increase in registrations of these cancers. The visibility of skin cancers might have lead to seasonal variation in rapidity of presentation to medical care, for instance for social reasons, or the results might reflect a short induction period effect of exposure to a seasonal insult, perhaps sun radiation, on the aetiology, growth or symptoms of skin cancers; for melanoma there is previous evidence suggesting a short induction period aetiological effect of sun radiation.


Br. J. Cancer (1985), **52**, 893–900

### Seasonality of presentation of cutaneous melanoma, squamous cell cancer and basal cell cancer in the Oxford Region

A.J. Swerdlow*

*University of Glasgow, Department of Community Medicine, Ruchill Hospital, Glasgow G20 9NB, Scotland, UK.*

**Summary** The seasonality of presentation of 1019 skin melanomas in Oxford Region 1952–1975, and of 1,523 squamous cell and 4,865 basal cell skin cancers in the region 1967–1975, were analysed using data from the Oxford Cancer Registry. For males and for females, for each of the histologies there was a peak of presentations during July to September. In further subdivisions of the data by age and by skin site, a summer or autumn peak was generally present except where numbers of cases were small. Amplitude of seasonality did not show consistent differences by histology, sex, or skin site, but for both melanoma and squamous cell cancer amplitude was greater for persons aged under 55 years than for older persons. There was no substantial seasonality for presentations of cancers of all non-skin sites in the region. The seasonality of presentation of skin cancers appeared not to be mainly an artefact of the cancer registration process or of organisational aspects of medical care attendance, and only a small proportion of it could be explained as an artefact of the longer term increase in registrations of these cancers. The visibility of skin cancers might have lead to seasonal variation in rapidity of presentation to medical care, for instance for social reasons, or the results might reflect a short induction period effect of exposure to a seasonal insult, perhaps sun radiation, on the aetiology, growth or symptoms of skin cancers; for melanoma there is previous evidence suggesting a short induction period aetiological effect of sun radiation.

Several recent studies (Fears *et al.*, 1977; Houghton *et al.*, 1978; Wigle, 1978; Swerdlow, 1979; Houghton *et al.*, 1980; Houghton & Viola, 1981; MacKie & Aitchison, 1982) have suggested that cutaneous malignant melanoma incidence may have a short induction period after exposure to sun radiation. One finding which could be interpreted as supporting this hypothesis is the seasonal pattern of first diagnosis of melanoma found in one or both sexes in Sweden (Malec & Eklund, 1978), the Third National Cancer Survey (TNCS) areas of the United States (Scotto & Nam, 1980), Hawaii (Hinds *et al.*, 1981) and Western Australia (Holman & Armstrong, 1981), and perhaps in Lane County, Oregon (Morton & Starr, 1979) – such seasonality of presentation could reflect real seasonality of incidence, which could most readily be explained by a short induction period from a seasonal aetiological insult such as ultraviolet radiation exposure. However, several alternative explanations of the seasonal presentation are possible and have not been fully explored in previous work: the disease might be noticed more rapidly in summer and hence present more often then; or the way in which medical care is organised or used might cause seasonality of medical appointments and hence of first diagnoses; or imperfections of the registration process or seasonal variations in the size of population at risk might lead to apparent seasonality; or seasonality could occur as an artefact of an increasing or a decreasing secular trend. The present study investigated seasonality of first attendance of skin melanoma cases in the Oxford Health Region of England, compared this with the seasonal pattern of presentations for the two other main histologies of skin cancer – basal and squamous cell cancers – in the region, and investigated the plausibility of various explanations for the seasonality.

*Present address: Office of Population Censuses and Surveys, St Catherines House, 10 Kingsway, London WC2B 6JP.

Received 7 March 1985; and in revised form, 3 September 1985.

### Materials and methods

The Oxford Cancer Registry has collected data on cancers in residents of the Oxford region since 1952.* Registration of cancers is voluntary. The data sources and methods used by the registry have been detailed by Hunt (1976). Completeness of ascertainment and accuracy of registration, as

*Since 1952 data have been collected on patients treated in hospitals in the Region, and since 1957 also on patients treated outside the region or not attending hospital. Small changes were made to the boundaries of the Region on 1st January 1974. These alterations in coverage are very unlikely to have affected the results materially.

© The Macmillan Press Ltd., 1985

judged from various indirect measures, appear generally to be high (Doll *et al.*, 1970; Waterhouse *et al.*, 1976, 1982; Scott, 1983).

Data were extracted from the files of the Oxford Cancer Registry on the age, sex, histology, tumour site and date of first attendance (first hospital attendance for the present tumour or, if no hospital attendance had occurred, first medical presentation) of all skin melanomas registered incident in residents of the Oxford Region*, 1952–75. Coding of skin site was by the Eighth Revision of the International Classification of Diseases (World Health Organization, 1967), with the earlier years of data recoded to this revision. Data on the same variables were extracted for all other skin tumours registered incident 1967–75 in the region; lack of computerisation of the registry made it impractical to extract data for squamous and basal cell cancers for earlier years. Data were also extracted for all non-skin tumours registered incident 1970–72; again, lack of computerisation limited the years of data available.

Harmonic seasonality of first attendance was tested by the method of Edwards (1961) when this method was applicable. Edwards' test is inappropriate if the sample size is less than 50 (Hewitt *et al.*, 1971) or if the seasonal data do not fit a harmonic curve. The fit of the data to a simple harmonic curve was therefore tested (Walter & Elwood, 1975), and where the data were significantly non-harmonic or the sample size was less than 50 the non-parametric method of Hewitt *et al.* (1971) was used to test significance of seasonality. Where more than 6 of the monthly values are zero, the test of Hewitt *et al.* (1971) is also inappropriate and no test was undertaken.

In order to test whether it was likely that potential artefacts of the registration process, seasonal variation in the population at risk, and organisational aspects of medical care attendance were of importance, or secular trends of incidence were of importance, the numbers of skin cancers expected in each month on the basis of these artefacts were first estimated for each sex for each histology, and then the potential effects of these expected seasonal variations on actual seasonality of presentation were assessed as described below. For estimation of the possible effects of the registration process, variation in the population at risk, and organisational aspects of medical care attendance, the expected numbers were obtained by taking monthly totals of first presentations of all patients with non-skin cancers, by sex, in the Oxford Region in 1970–1972 as a surrogate for these effects. For assessment of the extent to which secular trends of incidence were responsible for seasonality, the expected numbers were obtained from linear regression of incidence on year. The seasonality which each of these sets of expected numbers gave on Edwards' test was then calculated, and compared to the seasonality obtained by applying Edwards' test to the actual incidence data for the corresponding sex and histology of skin cancer. Also, for each sex for each histology, seasonality of actual presentations of skin cancers was re-calculated by the method of Walter and Elwood (1975) allowing for the numbers of cases expected from non-skin cancer presentations, and then allowing for linear regression expectations.

### Results

Three hundred and fifty-nine melanomas of the skin were incident in males and 668 in females in the Oxford Region, 1952–1975. One thousand and sixty five squamous cell cancers of the skin in males and 461 in females, and 2,661 basal cell skin cancers in males and 2,221 in females were incident in the region 1967–1975. The month of first attendance (Table I) was known for 98.3% (353) of males and 99.7% (666) of females with melanoma, 99.8%

**Table I** Month of presentation of malignant melanoma of the skin, squamous cell and basal cell skin cancers, by sex, Oxford Region.

| *Histology, and years of data* | *Sex* | *No. of cases by month of presentation* | | | | | | | | | | | | | |
|---|---|---|---|---|---|---|---|---|---|---|---|---|---|---|---|
| | | *Jan.* | *Feb.* | *Mar.* | *Apr.* | *May* | *June* | *July* | *Aug.* | *Sept.* | *Oct.* | *Nov.* | *Dec.* | *Not known* | *Total* |
| Malignant melanoma 1952–1975 | Males | 33 | 31 | 23 | 29 | 27 | 29 | 29 | 34 | 36 | 29 | 30 | 23 | 6 | 359 |
| | Females | 41 | 54 | 57 | 47 | 71 | 59 | 61 | 54 | 55 | 62 | 49 | 56 | 2 | 668 |
| Squamous cell cancer 1967–1975 | Males | 83 | 74 | 95 | 87 | 81 | 98 | 92 | 91 | 99 | 94 | 97 | 72 | 2 | 1,065 |
| | Females | 40 | 21 | 38 | 32 | 32 | 41 | 47 | 44 | 50 | 32 | 41 | 42 | 1 | 461 |
| Basal cell cancer 1967–1975 | Males | 210 | 194 | 184 | 183 | 213 | 222 | 242 | 236 | 255 | 270 | 269 | 178 | 5 | 2,661 |
| | Females | 183 | 156 | 154 | 142 | 180 | 206 | 208 | 202 | 197 | 219 | 215 | 147 | 12 | 2,221 |

(1,063) of males and 99.8% (460) of females with squamous cell cancer, and 99.8% (2,656) of males and 99.5% (2,209) of females with basal cell cancer. All seasonality testing was based on these patients. The site distribution of their tumours is given in Table II; it is virtually the same as the distribution for all incident cases – all percentages in the table are within one per cent of the corresponding percentages for all incident cases. Melanomas in males were mainly on the trunk and lower limbs; in females about half were on the lower limbs. In each sex, over half of the squamous cell cancers and over 80 per cent of the basal cell cancers were on the face.

Tables III & IV show the results of testing the seasonality of presentation of the skin cancers, by histology and site, for females and for males. Where a cell in these tables is based on less than 50 cases or the data did not fit a harmonic curve, the results of non-parametric testing of the significance of seasonality and the season of peak incidence (taken as the six-month period giving the highest ranking score) are shown in brackets, and the amplitude of seasonality, for which no non-parametric test was available, is not given. For each sex for each of the three histologies, the peak incidence for all skin sites combined was during July to September; indeed, with the exception of melanoma in females,

**Table II** Incidence of malignant melanoma of the skin, squamous cell and basal cell skin cancers of known month of presentation, by anatomic site, Oxford Region.

| | *Malignant melanoma 1952–1975* | | *Squamous cell 1967–1975* | | *Basal cell 1967–1975* | |
|---|---|---|---|---|---|---|
| *Skin site* | *Males* *n* (%) | *Females* *n* (%) | *Males* *n* (%) | *Females* *n* (%) | *Males* *n* (%) | *Females* *n* (%) |
| Face | 65 (18) | 95 (14) | 737 (69) | 259 (56) | 2,206 (83) | 1,871 (85) |
| Scalp and neck | 29 (8) | 26 (4) | 84 (8) | 37 (8) | 210 (8) | 175 (8) |
| Trunk | 107 (30) | 86 (13) | 46 (4) | 41 (9) | 146 (5) | 118 (5) |
| Upper limb | 55 (16) | 115 (17) | 151 (14) | 59 (13) | 60 (2) | 17 (1) |
| Lower limb | 97 (27) | 343 (52) | 40 (4) | 64 (14) | 29 (1) | 26 (1) |
| Multiple site and site not specified | 0 (0) | 1 (0) | 5 (0) | 0 (0) | 5 (0) | 2 (0) |
| Total | 353 (100) | 666 (100) | 1,063 (100) | 460 (100) | 2,656 (100) | 2,209 (100) |

**Table III** Seasonality[c] of presentation of malignant melanoma, squamous cell and basal cell skin cancers in females, by skin site, Oxford Region.

| | *Malignant melanoma 1952–1975* | | *Squamous cell cancer 1967–1975* | | *Basal cell cancer 1967–1975* | |
|---|---|---|---|---|---|---|
| *Skin site* | *Month of peak (or 6 months with maximum ranking score in non-parametric test)* | *Amplitude* | *Month of peak (or 6 months with maximum ranking score in non-parametric test)* | *Amplitude* | *Month of peak (or 6 months with maximum ranking score in non-parametric test)* | *Amplitude* |
| Face | October | 0.45[b] | August | 0.24[a] | (June–November)[ad] | — |
| Scalp and neck | (December–May) | — | (August–January) | — | July | 0.15 |
| Trunk | June | 0.16 | (August–January) | — | (July–December)[d] | — |
| Upper limb | April | 0.14 | July | 0.22 | —[e] | — |
| Lower limb | July | 0.14 | (August–January)[d] | — | (July–December)[a] | — |
| All sites | July | 0.10 | September | 0.18[a] | September | 0.17[b] |

[a]$P<0.05$; [b]$P<0.01$; [c]Seasonality tested by Edwards (1961) test, except for results in brackets which are by the non-parametric test of Hewitt *et al.* (1971). The non-parametric test was employed where cells were based on less than 50 cases, except for cells marked [d] where the test was employed because the seasonal data did not fit a harmonic curve; [e]More than two 6 month intervals each gave the maximum ranking score in the non-parametric test.

**Table IV** Seasonality[c] of presentation of malignant melanoma, squamous cell and basal cell skin cancers in males, by skin site, Oxford Region.

| Skin site | *Malignant melanoma 1952–1975* Month of peak | Amplitude | *Squamous cell cancer 1967–1975* Month of peak (or 6 months with maximum ranking score in non-parametric test) | Amplitude | *Basal cell cancer 1967–1975* Month of peak (or 6 months with maximum ranking score in non-parametric test) | Amplitude |
|---|---|---|---|---|---|---|
| Face | June | 0.29 | July | 0.16[a] | September | 0.17[b] |
| Scalp and neck | —[d] | — | December | 0.26 | July | 0.19 |
| Trunk | September | 0.23 | (April–September) | — | August | 0.32[a] |
| Upper limb | October | 0.05 | October | 0.10 | August | 0.23 |
| Lower limb | December | 0.12 | (October–March) | — | (June–November) | — |
| All sites | September | 0.09 | August | 0.09 | September | 0.17[b] |

[a]$P<0.05$; [b]$P<0.01$; [c]Seasonality tested by the method of Edwards (1961) except for results in brackets for which the non-parametric test of Hewitt *et al.* (1971) was employed because the cells were based on less than 50 cases; [d]More than two 6-month intervals each gave the maximum ranking score in the non-parametric test.

for which the peak was in July, all peaks were within approximately one month from mid-August to mid-September. For individual skin sites there was greater variation in the month of peak incidence, as would be expected with the small sample size in many of the cells. However, the peaks for cells with more than 50 cases were generally in late summer or autumn, all significant peaks were during July to September (or on non-parametric testing the mid-point of the maximum six months was between July and September), and there was no evidence of a systematic tendency for any site, sex, or histology to peak at any other season. Amplitude of seasonality showed no evidence of systematic variation by site, sex, or histology. In particular, the amplitude was not greater for melanoma than for the other histologies (indeed, for each sex, for all sites combined the amplitude for melanoma was less than that for either of the other two histologies), and the amplitude for melanoma of intermittently exposed skin sites was not greater than the amplitude for melanoma of permanently exposed sites (for each sex, the greatest amplitude was actually for the face).

Examination of the data divided into two age groups, under 55 years and 55 years and above [following the age-grouping used in a previous study by Scotto and Nam (1980)], gave no evidence of systematic differences by age in the peak season of seasonality, either for all sites combined (Table V) or for individual sites (not shown in the Table). However, for melanoma and for squamous cell cancer amplitude of seasonality was substantially greater in persons under 55 years of age than in older persons.

For malignancies of all non-skin sites incident in the Oxford Region 1970–1972 there was no significant seasonality of presentation in either sex (for males $n=8{,}662$, amplitude$=0.03$, $P=0.25$; for females $n=8{,}702$, data significantly non-harmonic, no significant seasonality on non-parametric testing). In each sex, the lowest monthly total was in December and the total for January was particularly high (January had the highest monthly total for females, the fourth highest for males). When seasonality of skin cancer presentation was re-tested, for each sex for each histology, with adjustment for the seasonal variation which would be expected on the basis of non-skin cancer presentations for the same sex (as a surrogate for possible effects of the registration process, seasonal variation in the population at risk, and organisational aspects of medical care attendance) there were no substantial changes compared to the unadjusted seasonality findings presented above – the angle of peak changed by 12 degrees or less (i.e. by less than half a month), the amplitude for males decreased by from 0.03 to 0.04, and the amplitude for females was unchanged or increased by up to 0.03.

The number of cases of each histology presenting per annum increased in each sex during the study period. However, application of Edwards' test to the monthly expected numbers from linear regression of incidence on year, for each sex for each histology, gave amplitudes of seasonality varying from 0.01 to 0.03 – far less than the

**Table V** Seasonality[c] of presentation of melanoma, squamous cell and basal cell skin cancers by age-group, Oxford Region.

| | *Malignant melanoma 1952–1975* | | *Squamous cell cancer 1967–1975* | | *Basal cell cancer 1967–1975* | |
|---|---|---|---|---|---|---|
| *Age-group* | *Month of peak* | *Amplitude* | *Month of peak* | *Amplitude* | *Month of peak (or 6 months with maximum ranking score in non-parametric test)* | *Amplitude* |
| *Male* | | | | | | |
| <55 years | October | 0.20 | April | 0.23 | September | 0.17[a] |
| ≧55 years | June | 0.15 | August | 0.11[a] | September | 0.17[b] |
| *Female* | | | | | | |
| <55 years | June | 0.19[a] | September | 0.35 | October | 0.13 |
| ≧55 years | October | 0.08 | September | 0.18[a] | (June–November[a])[d] | — |

[a]$P<0.05$; [b]$P<0.01$; [c]Seasonality tested by the method of Edwards (1961), except for results in brackets which are by the non-parametric test of Hewitt *et al.* (1971); [d]Seasonal distribution of cases significantly non-harmonic.

amplitudes which had been found using the actual monthly incidence data; in all instances the peak month from the seasonality test was September and the data were not a significantly bad fit to a harmonic curve. The peak and amplitude of seasonality of actual skin cancer presentations calculated allowing for expectations from linear regression were not, for any histology in males or in females, substantially different from the peak and amplitude for unadjusted skin cancer seasonality; the angle of peak altered by 10 degrees at most (except for basal cell cancer in females, for which the data were significantly incompatible with a simple harmonic curve, and the test was therefore not valid) and the amplitude remained unaltered or decreased by at most 0.03.

### Discussion

The skin site distribution of cutaneous melanoma in the Oxford Region is very similar to that in many other registry-based studies of white populations (Jensen & Bolander, 1980; Crombie, 1981; Muir & Nectoux, 1981; Swerdlow, 1984). The seasonality data from Oxford add to the evidence that there is a marked seasonal pattern, with a summer peak, to cutaneous melanoma presentations in white populations (Malec & Eklund, 1978; Morton & Starr, 1979; Scotto & Nam, 1980; Hinds *et al.,* 1981; Holman & Armstrong, 1981) although one previous study (Elwood & Gallagher, 1983) did not find such seasonality. One investigation (Holman *et al.,* 1983*a*) has presented data which suggest that the degree of seasonality may vary between histologic sub-types of melanoma; in the present study, data by histologic sub-type were not available to investigate this. There appear to have been no studies published previously of monthly seasonality of presentation of non-melanoma histologies of skin cancer. The findings in the present study on seasonality for non-melanoma skin cancers clearly require re-testing in other populations, but the high levels of significance of seasonality in several instances in the Oxford data indicate that it is very improbable that the results are attributable to chance.

Several potential artefacts could lead to seasonality of skin cancer presentation. Organisational aspects of medical care attendance might cause seasonality of hospital appointments: for instance, clinics may be closed or patients away from home over holiday periods, or the delay between first consultation with a general practitioner and first hospital attendance might vary seasonally according to the workload of the medical staff involved. Artefacts of the registration process might also cause apparent seasonality: for instance, diagnostic recording or cancer registration might deteriorate at times when staff workload was particularly high. A further potential source of apparent seasonality would be any seasonal variation in the size of the population at risk. If artefacts of the registration process or of organisational aspects of medical care attendance or of the size of the population at risk were present, they might well apply to non-skin cancers as well as to skin cancers. It appears likely, indeed, that one such artefact at least did occur for non-skin cancers: the comparatively low number of presentations in December and high number in January in each sex may well have occurred because clinics were closed and patients reluctant to consult over Christmas to New Year. However, as in the United States (Scotto & Nam, 1980) and Hawaii (Hinds *et al.*, 1981), there was in Oxford region no significant harmonic seasonality of presentation of non-skin tumours. Furthermore, adjustment of the Oxford skin cancer data for seasonal variation in presentation of non-skin cancers (as a surrogate for registration, organisational aspects of medical care attendance, and population size effects) did not alter substantially the seasonality findings for skin cancers. Although in any one population or registry it is possible that the above artefacts might exist for skin melanomas but not for non-skin cancers, it seems unlikely that this same specificity would have occurred in several different countries. Complete registration of non-melanoma skin cancers is particularly difficult (Waterhouse *et al.*, 1976; Scotto & Fraumeni, 1982), and because of the infrequency of fatality from such tumours they might be given relatively low priority for hospital appointments and hence be particularly likely to suffer delays between general practitioner consultation and first hospital attendance. Although there is no reason to believe that any incompleteness of registration of skin cancers or delay in hospital appointments in the Oxford region did in fact substantially vary systematically by season, these remain possibilities requiring further investigation.

The visibility and direct accessibility of skin cancers are differences from cancers generally which might lead to seasonality of presentation (through seasonal differences in rapidity of recognition of tumours and/or psychological or social factors affecting rapidity of presentation) not shared by other cancers generally. Some data are available, however, which are not those which would be expected if seasonal differences in dress and hence in skin visibility led to more rapid presentation of tumours in summer than in winter: seasonality of presentation of melanomas has been found in Hawaii where clothing styles are the same the year round (Hinds *et al.*, 1981); thickness of melanomas in British Columbia (Elwood & Gallagher, 1983) did not vary significantly by site or by sex (assessed separately for nodular and for superficial spreading tumours) suggesting that presentation was not more rapid for tumours on more exposed skin (this was, however, in a study which found no seasonality of month of diagnosis either); and in the present study the amplitude of seasonality of skin cancer presentations was generally no less for face tumours, whose visibility is not affected by seasonal variation in dress, than for trunk and limb sites, whose visibility is so affected. Nevertheless, potential seasonal differences in rapidity of presentation remain a possibility needing investigation more directly.

Seasonality would also be indicated on Edwards' (1961) test or on the test of Hewitt *et al.* (1971) if there were an increasing (or a decreasing) secular trend in the numbers of cases registered per month: if incidence was increasing, the number of cases occurring per month early in a year would tend on average to be lower than in the later months of the same year (or, if incidence was decreasing there would tend to be more cases in early months than in late months of a year). Hinds *et al.* (1981) noted that the average rate of increase in melanoma diagnoses in Hawaii during their study would have given a relatively small difference between January–February and November–December numbers of cases, and they did not correct for it in their analysis. Other studies have not examined the effect of secular trends on seasonality findings for melanoma presentations. Calculation of seasonality of presentation of skin cancers in Oxford region allowing for linear trends in incidence showed only a modest reduction in amplitude of seasonality and virtually no change in peak compared to the results without such allowance, suggesting that linear trends were not the principal explanation of the study findings. For each sex and histology the correlation coefficient for a linear model of the secular trend was significant at $P<0.05$ (in three instances at $P<0.001$), suggesting that linear descriptions of the incidence data were satisfactory for the present purpose, and that fitting of more complex models would have been unlikely to have altered the conclusions.

A further possibility is that seasonality might reflect a short induction period effect of a seasonal insult on aetiology or growth or symptoms of skin tumours. For a seasonal insult to result in clear seasonality of presentation, the duration from the insult to presentation would need not to show great variability between individuals (by implication it would therefore need to be short) or to show substantial systematic seasonal variation. In the present study, data were not available to investigate this satisfactorily. It would help to clarify whether a seasonal insult could be responsible for seasonality of presentation, or whether factors affecting rapidity of presentation were important, if investigations were conducted into the degree of seasonality and of variability existing for the intervals between first symptom, first presentation to a general practitioner, and first hospital presentation, and the reasons for presentation at the point at which presentation occurred.

For cutaneous melanoma, the hypothesis that a short induction period aetiological effect is responsible for seasonality of presentation is supported by the finding by Holman *et al.* (1983*b*) in Western Australia that skin naevi excised in summer were more likely to have a junctional component and evidence of inflammatory response than those excised in winter.

Sun radiation exposure is at present the most plausible candidate for a short induction period insult causing seasonality of melanoma presentation because it is appropriately seasonal, there is strong evidence that it has a major role in melanoma aetiology (Elwood & Hislop, 1982; Lee, 1982; MacKie, 1983; Swerdlow, 1984), and there is evidence that it has a short induction period aetiological effect (Fears *et al.,* 1977; Houghton *et al.,* 1978; Wigle, 1978; Swerdlow, 1979; Houghton *et al.,* 1980; Houghton & Viola; 1981; MacKie & Aitchison, 1982). Other seasonal exposures could give alternative explanations if they were shown likely to be major aetiological factors for melanoma, but there is no other exposure for which there is currently strong evidence that it is both a major risk factor and shows seasonal variation. Diet, for instance, varies seasonally, but there is no strong evidence that it is aetiological. Cohen (1983) has suggested that a hormonal effect might be related to seasonality. He noted that the female to male ratio for melanoma diagnoses was greater in summer than in winter in published US (Scotto & Nam, 1980) and Swedish (Malec & Eklund, 1978) data, and hypothesised that this might reflect an endogenous seasonality of female sex hormone balance affecting melanoma growth. In the present data the female to male ratio showed some indication of a summer peak for melanoma under age 55 years, but not clearly for melanoma at older ages (or for squamous or basal cell cancers at young or old ages). Whilst compatible with the hormonal hypothesis, seasonality of the sex ratio of melanoma presentations could equally reflect differences in sun exposure between the sexes, and this seems a more likely explanation because of the lack of strong evidence that endogenous hormones are a major risk factor for melanoma.

Findings on the seasonality of melanoma presentation by age and by skin site have not been consistent between different studies. With regard to seasonality by age, Scotto and Nam (1980) found that the greatest seasonality was for females under 55 years of age, and in the present study, too, seasonality was greater for this age group than for older subjects. Hinds *et al.* (1981), however, found seasonality greater for persons aged 50 years and over than for younger persons. Scotto and Nam (1980) found seasonality particularly pronounced for the upper and lower extremities in females and for the upper extremities in males. Hinds *et al.* (1981), using data for both sexes combined, found seasonality particularly pronounced for the head and neck and for the lower extremities. In the present study the amplitude of seasonality in each sex was greatest for the face and least for the limbs. Malec and Eklund (1978) did not analyse their data by a monthly seasonality test, but commented on the particular seasonality for lower limbs.

Scotto and Nam (1980) proposed that their site- and sex-specific findings could be explained if promotion of tumour growth occurred from short periods of high intensity UV-B exposure, and Hinds *et al.* (1981) suggested that the seasonality found for male as well as for female lower limbs in Hawaii fitted this hypothesis, since males commonly wear shorts in Hawaii. The particular seasonality found for melanoma of the face, not for melanoma of intermittently exposed sites, in the present study is not the result which would obviously be expected from the hypothesis; no firm conclusion can be drawn, however, because of the relatively small numbers on which many of the site-specific calculations were based and the lack of site-specific exposure data. The Oxford region population probably differs substantially in pattern and degree of sun radiation exposure from the other populations for which statistical analyses of seasonality of melanoma presentations by skin site have been published (the TNCS areas of the United States, and Hawaii); the differences between the populations in site- and sex-specific seasonality of melanoma presentations may be related to these exposure differences, but objective exposure data are needed to test this.

Whilst there is substantial evidence that non-melanoma skin cancer incidence is aetiologically related to cumulative exposure to ultraviolet radiation (Scotto & Fraumeni, 1982), there does not appear to be any previous evidence, nor any epidemiological test of the possibility, of an additional, short induction period effect of ultraviolet radiation on incidence of these tumours. The long history of the lesion often obtained clinically at presentation of non-melanoma skin cancers, especially basal cell cancers, makes it at first sight less likely that if there is seasonality of incidence of these tumours it would manifest as seasonality of presentation, and thus makes it more likely that seasonality of presentation may have been due to seasonal variation in rapidity of presentation (for instance for social or psychological reasons); both alternatives, however, remain possible. If seasonality of non-melanoma skin cancer presentations were shown to be due to factors causing seasonal variation in rapidity of presentation, it would particularly need investigation whether these factors were responsible also for cutaneous melanoma presentation seasonality.

The seasonality of presentation of skin tumours and the reasons for such seasonality need further investigation. If seasonal variation in rapidity of presentation were shown, useful information might be gained about the reasons for delays in presentation and hence ways in which to minimise these; if a short induction period effect were shown, this might well have important implications for prevention of the tumours.

I thank Miss C. Hunt and her staff at Oxford Cancer Registry for registry data, Mrs K. H. Wood and Mrs F. Garven for programming of Walter and Elwood's test, Mr D. Hole for advice about the test, Dr M. R. Alderson and Professor J. M. Elwood for helpful comments on drafts of the manuscript, and Ms L. Semke for secretarial help.

### References

COHEN, P. (1983). Cancer and seasonal patterns. *Am. J. Epidemiol.*, **118**, 785.

CROMBIE, I.K. (1981). Distribution of malignant melanoma on the body surface. *Br. J. Cancer*, **43**, 842.

DOLL, R., MUIR, C. & WATERHOUSE, J., (eds) (1970). *Cancer Incidence in Five Continents*. Volume II. Springer-Verlag (for the UICC): Berlin.

EDWARDS, J.H. (1961). The recognition and estimation of cyclic trends. *Ann. Hum. Genet.*, **25**, 83.

ELWOOD, J.M. & GALLAGHER, R.P. (1983). Site distribution of malignant melanoma. *Canad. Med. Assn. J.*, **128**, 1400.

ELWOOD, J.M. & HISLOP, T.G. (1982). Solar radiation in the etiology of cutaneous malignant melanoma in Caucasians. *Natl Cancer Inst. Monogr.*, **62**, 167.

FEARS, T.R., SCOTTO, J. & SCHNEIDERMAN, M.A. (1977). Mathematical models of age and ultraviolet effects on the incidence of skin cancer among whites in the United States. *Am. J. Epidemiol.*, **105**, 420.

HEWITT, D., MILNER, J., CSIMA, A. & PAKULA, A. (1971). On Edwards' criterion of seasonality and a non-parametric alternative. *Br. J. Prev. Soc. Med.*, **25**, 174.

HINDS, M.W., LEE, J. & KOLONEL, L.N. (1981). Seasonal patterns of skin melanoma incidence in Hawaii. *Am. J. Pub. Health*, **71**, 496.

HOLMAN, D. & ARMSTRONG, B. (1981). Re: 'skin melanoma and seasonal patterns'. *Am. J. Epidemiol.*, **113**, 202.

HOLMAN, C.D.J., ARMSTRONG, B.K. & HEENAN, P.J. (1983*a*). A theory of the etiology and pathogenesis of human cutaneous malignant melanoma. *J. Natl Cancer Inst.*, **71**, 651.

HOLMAN, C.D.J., HEENAN, P.J., CARUSO, V., GLANCY, R.J. & ARMSTRONG, B.K. (1983*b*). Seasonal variation in the junctional component of pigmented naevi. *Int. J. Cancer*, **31**, 213.

HOUGHTON, A.N. & VIOLA, M.V. (1981). Solar radiation and malignant melanoma of the skin. *J. Am. Acad. Dermatol.*, **5**, 477.

HOUGHTON, A., FLANNERY, J. & VIOLA, M.V. (1980). Malignant melanoma in Connecticut and Denmark. *Int. J. Cancer*, **25**, 95.

HOUGHTON, A., MUNSTER, E.W. & VIOLA. M.V. (1978). Increased incidence of malignant melanoma after peaks of sunspot activity. *Lancet*, **i**, 759.

HUNT, C. (1976). UK, England, Oxford Region. In: *Cancer Incidence in Five Continents*, Waterhouse *et al.* (eds) Vol. III, p. 380. IARC: Lyon.

JENSEN, O.M. & BOLANDER, A.M. (1980). Trends in malignant melanoma of the skin. *World Health Stat. Q.*, **33**, 2.

LEE, J.A.H. (1982). Melanoma and exposure to sunlight. *Epidemiol. Rev.*, **4**, 110.

MACKIE, R.M. (1983). The pathogenesis of cutaneous malignant melanoma. *Br. Med. J.*, **287**, 1568.

MACKIE, R.M. & AITCHISON, T. (1982). Severe sunburn and subsequent risk of primary cutaneous malignant melanoma in Scotland. *Br. J. Cancer*, **46**, 955.

MALEC, E. & EKLUND, G. (1978). The changing incidence of malignant melanoma of the skin in Sweden, 1959–1968. *Scand. J. Plast. Reconstr. Surg.*, **12**, 19.

MORTON, W.E. & STARR, G.F. (1979). Epidemiologic clues to the cause of melanoma. *Western J. Med.*, **131**, 263.

MUIR, C.S. & NECTOUX, J. (1981). Time trends: malignant melanoma of skin. In *Trends in Cancer Incidence. Causes and Practical Implications*, Magnus (ed) p. 365. Hemisphere Publishing Corporation: Washington.

SCOTT, A.J. (1983). Cancer registration. Report on data quality project. Office of Population Censuses & Surveys, unpublished.

SCOTTO, J. & FRAUMENI, J.F. Jr. (1982). Skin (other than melanoma). In *Cancer Epidemiology and Prevention*, Schottenfeld & Fraumeni (eds) p. 996. W.B. Saunders Company: Philadelphia.

SCOTTO, J. & NAM, J.-M. (1980). Skin melanoma and seasonal patterns. *Am. J. Epidemiol.*, **111**, 309.

SWERDLOW, A.J. (1979). Incidence of malignant melanoma of the skin in England and Wales and its relationship to sunshine. *Br. Med. J.*, **2**, 1324.

SWERDLOW, A.J. (1984). Epidemiology of cutaneous malignant melanoma. In *Clinics. Oncol.* Vol. **3**, no. 3, MacKie (ed) p. 407. W.B. Saunders Company: London.

WALTER, S.D. & ELWOOD, J.M. (1975). A test for seasonality of events with a variable population at risk. *Br. J. Prev. Soc. Med.*, **29**, 18.

WATERHOUSE, J., MUIR, C., CORREA, P. & POWELL, J., (eds) (1976). *Cancer Incidence in Five Continents*. Volume III. IARC: Lyon.

WATERHOUSE, J., MUIR, C., SHANMUGARTNAM, K. & POWELL, J. (eds) (1982). *Cancer Incidence in Five Continents*. Volume IV. IARC: Lyon.

WIGLE, D.T. (1978). Malignant melanoma of skin and sunspot activity. *Lancet*, **88**, 38.

WORLD HEALTH ORGANIZATION (1967). *Manual of the International Statistical Classification of Diseases, Injuries, and Causes of Death*. Eighth Revision. WHO: Geneva.